# Integrative Transcriptomic and Biochemical Profiling Reveals *Bacillus amyloliquefaciens* JL54 Primes *Larix olgensis* Defenses Against *Neofusicoccum laricinum* Attack

**DOI:** 10.3390/plants15081181

**Published:** 2026-04-11

**Authors:** Xiangyu Zhao, Fengze Yang, Lingyu Kong, Yanru Wang, Kexin Liu, Yinjuan Zhao, Xun Deng, Liwen Song, Ke Wei, Jiajin Tan

**Affiliations:** 1 Collaborative Innovation Center of Modern Forestry in South China, College of Forestry and Grassland, Nanjing Forestry University, Nanjing 210037, China; zhaoxy6472@163.com (X.Z.); yangfengze1005@163.com (F.Y.); 15862877156@163.com (L.K.); wangyanru@njfu.edu.cn (Y.W.); 13372039006@163.com (K.L.); zhaoyinjuan@njfu.edu.cn (Y.Z.); 2 Institute of Forestry Protection, Heilongjiang Forestry Academy, Harbin 150040, China; dxhappy@126.com; 3 Jilin Provincial Academy of Forestry Science, Changchun 130033, China; lwsong75@126.com; 4 Ecology and Nature Conservation Institute, Chinese Academy of Forestry, Beijing 100091, China; weike@caf.ac.cn

**Keywords:** biological control, induced resistance, endophytic bacteria, transcriptome, *Bacillus amyloliquefaciens*, *Neofusicoccum laricinum*

## Abstract

*Larix olgensis*, a keystone timber species in Northeast China, is increasingly threatened by *Neofusicoccum laricinum*-induced shoot blight, a devastating disease that compromises forest health and necessitates sustainable management strategies. Here, we demonstrate that the endophytic bacterium *Bacillus amyloliquefaciens* JL54 elicits multifaceted defense responses in *L. olgensis*, enhancing resistance to pathogen infection. Greenhouse assays revealed that JL54 pretreatment reduced disease incidence by 12.5% and achieved 43.75% control efficacy while maintaining host vigor. Histochemical analyses identified JL54-induced rapid hydrogen peroxide (H_2_O_2_) accumulation, extensive lignin deposition, and localized programmed cell death (PCD), indicative of a primed immune response. Transcriptomic analyses uncovered distinct temporal defense patterns: early-stage responses (0 h post-inoculation) were characterized by upregulation of cutin, suberin, and wax biosynthesis pathways, reinforcing physical barriers, whereas late-stage responses (12 h post-inoculation) were dominated by ribosome- and proteostasis-related pathways (e.g., heat shock proteins [HSPs], glutathione S-transferases [GSTs]) to mitigate cellular damage. Biochemical assays corroborated these findings, with JL54 colonization reducing membrane lipid peroxidation (27.2% decrease in malondialdehyde content) and significantly elevating the activity of key defense enzymes, including peroxidase (POD), phenylalanine ammonia-lyase (PAL), and GST. Phytohormone profiling implicated jasmonic acid (JA) as the central mediator of induced systemic resistance (ISR), with JL54-potentiated JA signaling preceding pathogen containment. Collectively, these results demonstrate that JL54 contributes to a coordinated defense strategy in *L. olgensis*, integrating structural reinforcement (cuticle/lignin), oxidative stress management, and JA-mediated immune priming. These insights advance the understanding of endophyte-conferred resistance in conifers and highlight JL54’s potential as a biocontrol agent for sustainable forestry.

## 1. Introduction

*Larix olgensis*, an economically significant timber species in northeastern China, is widely used in construction, shipbuilding, railway sleepers, and utility pole manufacturing due to its notable industrial value [1]. Larch forests also play critical ecological roles in water conservation, soil retention, and carbon sequestration, thereby contributing to oxygen release and ecosystem stability [2]. However, in recent years, these forests have been increasingly threatened by larch shoot blight. The disease spreads rapidly and induces premature tree decline and is caused by *Neofusicoccum laricinum* [3]. This pathogen disrupts the establishment of stable forest ecosystems and weakens ecological barrier functions [4]. Larch shoot blight is a highly dangerous infectious forest disease with severe destructive effects, which has been listed as a national forest plant quarantine pest in China for consecutive years (1980, 1996, 2004). By 2011, the damaged area of larch plantations caused by this disease had exceeded 50 hectares nationwide, inflicting substantial economic losses on the forestry industry. Current control measures predominantly rely on chemical fungicides such as chlorothalonil and thiophanate-methyl; however, prolonged application has resulted in increased pathogen resistance and heightened risks of environmental contamination. Consequently, the development of environmentally friendly and sustainable biocontrol strategies has become a critical priority.

The genus *Bacillus*, particularly *Bacillus amyloliquefaciens*, is a prominent group of plant endophytic bacteria. This species has garnered attention for its multifaceted plant-beneficial traits [5], including antagonism against phytopathogens, induction of systemic resistance, and promotion of plant growth [6,7,8]. For instance, *B. amyloliquefaciens* strain B4 has demonstrated efficacy in postharvest disease management by suppressing fungal pathogens through competitive colonization and antimicrobial metabolite production [9]. Furthermore, studies using *B. amyloliquefaciens* strains HSB1 and FZB42 revealed effective control of wolfberry (*Lycium* spp.) root rot alongside growth promotion in seedlings, while simultaneously modulating rhizobacterial community composition and enhancing the functional capacity of indigenous root-associated bacteria [10]. These examples highlight the potential of *B. amyloliquefaciens* as a versatile biocontrol agent; however, its application in conifer disease systems remains largely unexplored, and the specific mechanisms by which it exerts biocontrol effects in such systems remain largely unknown.

In elucidating the dynamic gene expression during host–pathogen interactions, transcriptome sequencing technology serves as a pivotal tool for dissecting plant disease resistance mechanisms. Previous studies have shown that pathogen-associated molecular patterns (PAMPs) are perceived by plant pattern-recognition receptors (PRRs), thereby activating PAMP-triggered immunity (PTI). For example, RNA-seq analysis of tomato plants infected with *Ralstonia solanacearum* identified 2698 significantly upregulated genes, which were primarily enriched in redox-related processes and defense-associated pathways [11]. Similarly, in *Gardenia jasminoides*, the accumulation of geniposide and crocin was positively correlated with the expression of *GPPPS* and *CCD* genes, under the regulation of MYB and WRKY transcription factors [12]. In rice (*Oryza sativa*), comparative transcriptomic analyses revealed that the resistance gene *Xa23* confers broad-spectrum resistance to bacterial blight caused by *Xanthomonas oryzae* pv. *oryzae* (Xoo), with 1645 differentially expressed genes associated with transcription factors, kinase-responsive genes, phenylpropanoid biosynthesis, and phytohormone signaling pathways [13].

Our laboratory previously isolated *B. amyloliquefaciens* JL54 from healthy *L. olgensis* tissues. This strain not only exhibits robust in vitro antagonism against *N. laricinum* [14], but also can stably colonize larch tissues and effectively reduce disease severity in pot trials [15]. Further field trials confirmed that JL54 achieved a 54% field control efficacy against larch shoot blight under natural conditions [16], fully verifying its potential as a biocontrol strain with practical application value in forestry production. However, the mechanistic basis of this protection—particularly its capacity to prime host defenses—remains unclear. Addressing this gap, the present study integrates histochemical, transcriptomic, and biochemical approaches to dissect JL54-induced resistance in *L. olgensis*.

## 2. Results

### 2.1. Inoculation with JL54 Confers Effective Protection Against N. laricinum in L. olgensis

**Figure 1:**
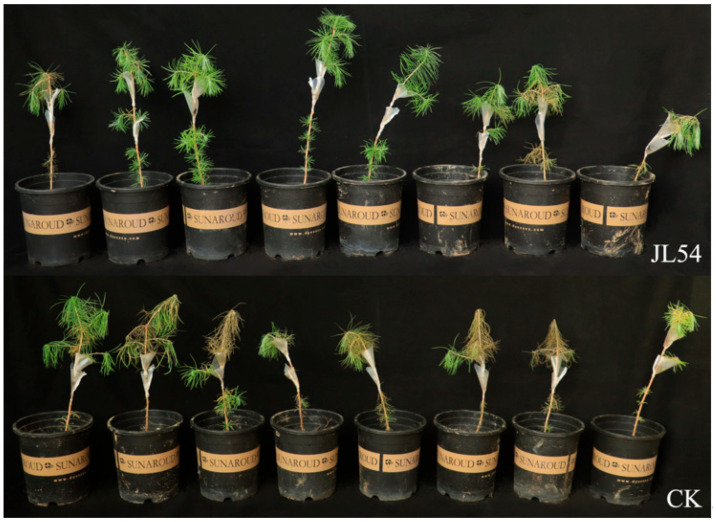
Biocontrol efficacy of *Bacillus amyloliquefaciens* JL54 against *Neofusicoccum laricinum*-induced shoot blight in *Larix olgensis* seedlings. Treatment groups: CK: Sterile water pretreatment 72 h prior to *N. laricinum* inoculation; JL54: JL54 bacterial suspension pretreatment 72 h prior to *N. laricinum* inoculation.

Compared with control seedlings (CK; sterile water pretreatment 72 h prior to *N. laricinum* inoculation), JL54-pretreated seedlings (JL54; JL54 bacterial suspension pretreatment 72 h prior to *N. laricinum* inoculation) exhibited reduced disease incidence and disease index in *L. olgensis* seedlings. In greenhouse assays, all CK seedlings developed disease symptoms by 16 days post-inoculation, whereas the JL54 group showed a lower disease incidence of 87.5% and a markedly reduced disease index (45 vs. 80 in the CK), corresponding to a control efficacy of 43.75%. In addition, JL54 group seedlings maintained green needles and upright stems, while CK plants displayed chlorotic needles, stem bending, and apical withering (Figure 1; Table 1).

### 2.2. JL54 Induces Immune Defense Responses in L. olgensis

**Figure 2:**
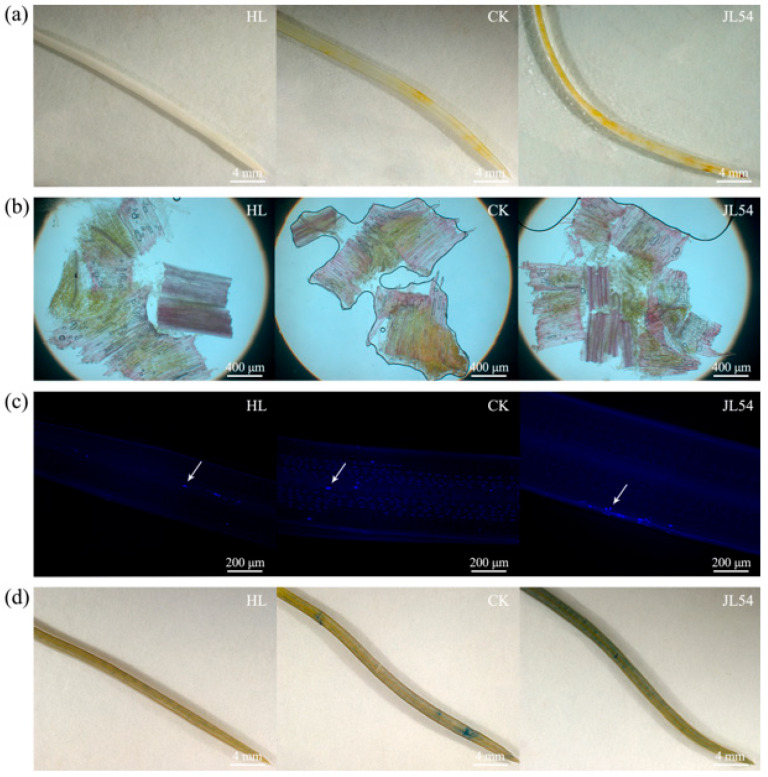
JL54 induces oxidative burst, cell wall reinforcement, and programmed cell death in *Larix olgensis* needles and stems: (**a**) H_2_O_2_ staining of needles (brown, scale bar = 4 mm); (**b**) lignin staining of stems (red, scale bar = 400 μm); (**c**) callose staining (blue areas indicated by arrows represent regions with concentrated callose deposition, scale bar = 200 μm); (**d**) Trypan blue staining of needles (blue, scale bar = 4 mm). Treatment groups: HL: Healthy controls (uninoculated); CK: Sterile water pretreatment 72 h prior to *N. laricinum* inoculation; JL54: JL54 bacterial suspension pretreatment 72 h prior to *N. laricinum* inoculation.

Histochemical analyses revealed distinct defense-associated responses in *L. olgensis* seedlings following JL54 treatment and pathogen challenge. Hydrogen peroxide (H_2_O_2_) staining in needles showed minimal yellow coloration in healthy control seedlings (HL; uninoculated), faint staining in pathogen-inoculated control plants, and intense yellow coloration in JL54-treated seedlings, indicating elevated H_2_O_2_ accumulation in response to JL54 pretreatment (Figure 2a). As a pivotal signaling molecule in plant immunity, H_2_O_2_ accumulation levels directly correlated with induced resistance.

Lignin staining of stem tissues demonstrated enhanced lignin deposition in both CK and JL54 groups compared with HL seedlings. Notably, JL54-treated seedlings exhibited the strongest red staining intensity, indicating higher lignin accumulation relative to the sterile water-treated control (Figure 2b). Lignin, a critical component of cell wall reinforcement during defense responses, directly contributed to resistance induction.

Callose staining showed that pathogen-challenged seedlings, including both control and JL54-treated groups, accumulated substantially more callose than HL seedlings. However, no obvious difference in callose deposition was observed between JL54 and CK seedlings following pathogen inoculation (Figure 2c).

Trypan blue staining revealed sparse blue-stained regions in HL seedlings, reflecting limited programmed cell death (PCD). In contrast, pathogen-inoculated control seedlings displayed scattered blue-stained areas, whereas JL54-treated seedlings exhibited larger and more contiguous blue-stained regions, indicating enhanced localized cell death following pathogen challenge (Figure 2d).

### 2.3. Transcriptomic Profiling Reveals Key Metabolic Pathways Regulated by JL54

**Figure 3:**
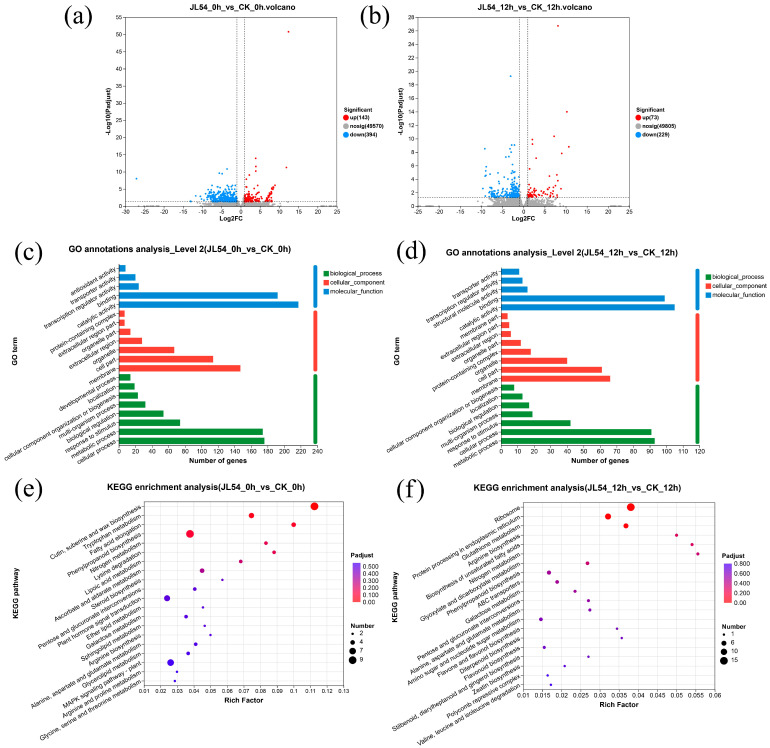
Transcriptomic analysis of *Larix olgensis* needles reveals *Bacillus amyloliquefaciens* JL54-mediated metabolic reprogramming under *Neofusicoccum laricinum* challenge: (**a**,**b**) Volcano plot of differentially expressed genes (DEGs) at 0 h post-inoculation (hpi) (JL54_0h vs. CK_0h) and 12 hpi (JL54_12h vs. CK_12h); |log_2_FC| ≥ 1, *p* < 0.05); dashed lines indicate significance thresholds. (**c**,**d**) GO annotation bar chart at 0 hpi and 12 hpi. (**e**,**f**) KEGG enrichment bubble plot at 0 hpi and 12 hpi. Treatment groups: CK_0h: CK (Sterile water pretreatment + *N. laricinum*) at 0 hpi; JL54_0h: JL54 (JL54 pretreatment + *N. laricinum*) at 0 hpi; CK_12h: CK (Sterile water pretreatment + *N. laricinum*) at 12 h; JL54_12h: JL54 (JL54 pretreatment + *N. laricinum*) at 12 h.

RNA-seq analysis demonstrated that JL54 pretreatment markedly altered the transcriptomic landscape of *L. olgensis* needles upon *N. laricinum* challenge (Figure 3a,b). At 0 h post-inoculation (hpi) (JL54_0h vs. CK_0h), a total of 537 differentially expressed genes (DEGs) were identified, including 143 upregulated and 394 downregulated genes. At 12 hpi (JL54_12h vs. CK_12h), 302 DEGs were detected, of which 73 were upregulated and 229 were downregulated.

Gene Ontology (GO) enrichment analysis demonstrated significant terms across both time points (Figure 3c,d), encompassing biological processes (BP), cellular components (CC), and molecular functions (MF). In the comparison group JL54_0h vs. CK_0h, differentially expressed genes were classified into 35 functional categories across the three GO domains, with 584 DEGs annotated to biological processes, 394 to cellular components, and 480 to molecular functions. In the comparison group JL54_12h vs. CK_12h, differentially expressed genes were classified into 30 functional categories across the three GO domains (Figure 3c,d), with 290 DEGs annotated to biological processes, 213 to cellular components, and 251 to molecular functions. The most abundantly annotated biological processes were cellular process (GO:0009987) and metabolic process (GO:0008152), which collectively represented the predominant functional categories.

Kyoto Encyclopedia of Genes and Genomes (KEGG) enrichment analysis revealed the top 20 significantly enriched pathways (Figure 3e,f). In the JL54_0h vs. CK_0h comparison group, 178 DEGs were annotated to 74 pathways, among which 8 pathways showed significant differences (*p* < 0.05). The most prominently enriched pathway was cutin, suberine and wax biosynthesis (map00073) with 9 genes, followed by fatty acid elongation (map00062, 4 genes), tryptophan metabolism (map00380, 5 genes), phenylpropanoid biosynthesis (map00940, 9 genes), lysine degradation (map00310, 3 genes), nitrogen metabolism (map00910, 3 genes), lipoic acid metabolism (map00785, 3 genes), and ascorbate and aldarate metabolism (map00053, 4 genes). The identified genes included 3-ketoacyl-CoA synthase, cytochrome proteins, dihydrolipoyl dehydrogenase, phenylalanine ammonia-lyase (PAL), SP12 protein, peroxidase (POD), and α-carbonic anhydrase. Cutin, suberin and wax biosynthesis, phenylpropanoid biosynthesis, and fatty acid elongation pathways are closely associated with the formation of plant structural barriers [17,18]. Enrichment in cutin, suberin and wax biosynthesis indicates activation of cuticular barrier formation, which may further strengthen the physical defense system of *L. olgensis*. Notably, the phenylpropanoid pathway included PAL and POD genes, which are key enzymes in lignin biosynthesis [19]. This result is consistent with the histochemical staining showing enhanced lignin deposition in JL54-treated seedlings (Figure 2b), suggesting that JL54 priming promotes cell wall reinforcement before pathogen invasion.

In the JL54_12h vs. CK_12h comparison group (Figure 3f), 87 DEGs were annotated to 38 pathways, with 7 pathways showing significant differences (*p* < 0.05). The most prominently enriched pathway was ribosome (map03010) with 15 genes, followed by protein processing in endoplasmic reticulum (map04141, 9 genes), glutathione metabolism (map00480, 6 genes), nitrogen metabolism (map00910, 2 genes), biosynthesis of unsaturated fatty acids (map01040, 2 genes), arginine biosynthesis (map00220, 2 genes), and glyoxylate and dicarboxylate metabolism (map00630, 3 genes). The identified gene products included 40S ribosomal small subunit proteins, 60S ribosomal large subunit proteins, SAD1-interacting factors, heat shock proteins (HSPs), glutathione S-transferases (GSTs), acyl-CoA desaturases, and glutamine synthetase. Enrichment of the glyoxylate and dicarboxylate metabolism and nitrogen metabolism pathways implied active maintenance of redox homeostasis and stress-related metabolic adjustments in the plants, which were likely closely associated with the H_2_O_2_ accumulation revealed by histochemical staining [20] (Figure 2a). Enrichment of the phenylpropanoid biosynthesis and unsaturated fatty acid biosynthesis pathways revealed sustained structural and membrane-related defense responses in the plants. This was further substantiated by the increased lignin deposition in the physiological assays, forming a defense barrier (Figure 2b). Notably, the enrichment of pathways related to endoplasmic reticulum protein processing and ribosome biogenesis indicated accelerated synthesis, folding, and repair of stress-related proteins. These cellular repair mechanisms are typically associated with oxidative stress responses and may serve as an important basis for the enhanced programmed cell death (PCD) observed in the JL54-treated groups [21] (Figure 2d).

### 2.4. Validation of Differentially Expressed Genes by RT-qPCR

**Figure 4:**
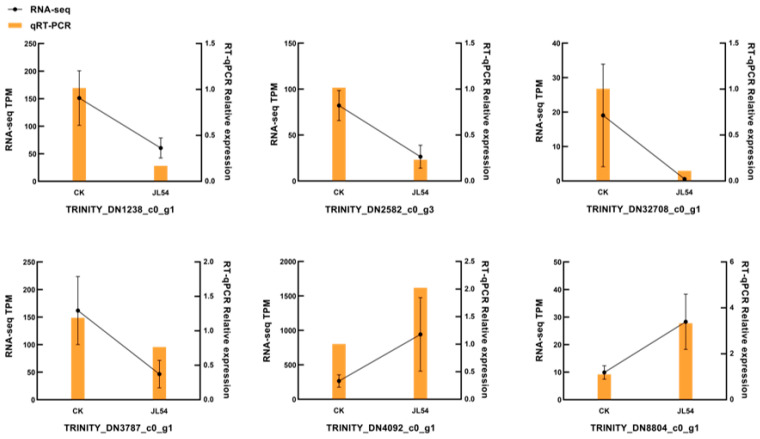
Validation of transcriptomic data by RT-qPCR analysis in *Larix olgensis* needles: Bar plots (RT-qPCR) and line plots (RNA-seq) show consistent expression trends for six selected DEGs (2 upregulated: *TRINITY_DN4092_c0_g1*, *TRINITY_DN8804_c0_g1*; four downregulated: *TRINITY_DN1238_c0_g1*, *TRINITY_DN2582_c0_g3*, *TRINITY_DN32708_c0_g1*, *TRINITY_DN3787_c0_g1*). Fold-change values (log_2_ scale) are normalized to the internal control *LoB80280*. Error bars indicate ±SE of three biological replicates. Treatment groups: CK: Sterile water pretreatment 72 h prior to *N. laricinum* inoculation; JL54: JL54 bacterial suspension pretreatment 72 h prior to *N. laricinum* inoculation.

To validate the accuracy of transcriptome data, six differentially expressed genes (2 upregulated and 4 downregulated) were randomly selected for reverse transcription quantitative polymerase chain reaction (RT-qPCR) verification. The selected genes represented key metabolic pathways, including protein processing in the endoplasmic reticulum (*TRINITY_DN4092_c0_g1*, *TRINITY_DN8804_c0_g1*) and glutathione metabolism (*TRINITY_DN1238_c0_g1*, *TRINITY_DN2582_c0_g3*, *TRINITY_DN32708_c0_g1*, *TRINITY_DN3787_c0_g1*). Despite differences in fold-change magnitudes between RT-qPCR and transcriptome data, both datasets showed consistent expression trends (Figure 4), validating the reliability of the RNA-seq results.

### 2.5. JL54 Enhances Defense Enzyme Activities to Bolster Disease Resistance in Larch

**Figure 5:**
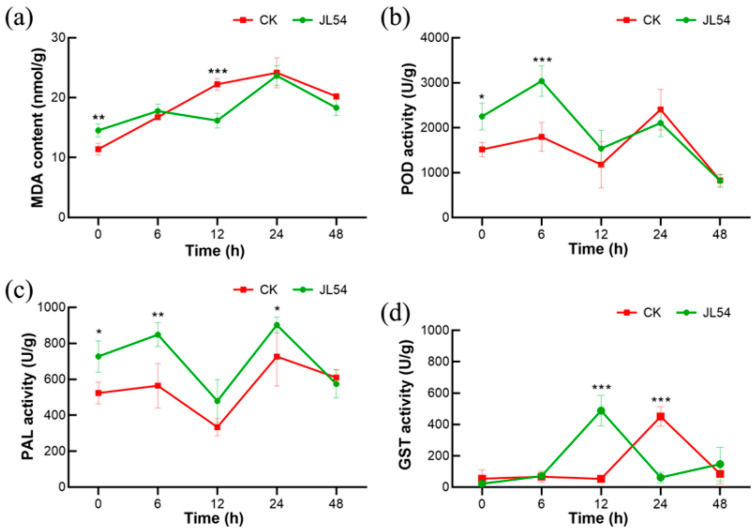
*Bacillus amyloliquefaciens* JL54 mitigates oxidative damage and primes defense enzyme cascades in *Larix olgensis* needles under *Neofusicoccum laricinum* challenge: (**a**) Malondialdehyde (MDA) content (nmol/g) at 0, 6, 12, 24, and 48 hpi. (**b**) Peroxidase (POD) activity (U/g) at 0, 6, 12, 24, and 48 hpi. (**c**) Phenylalanine ammonia-lyase (PAL) activity (U/g) at 0, 6, 12, 24, and 48 hpi. (**d**) Glutathione S-transferase (GST) activity (U/g) at 0, 6, 12, 24, and 48 hpi. Data are means ± SE of three biological replicates. Significant differences between treatments are indicated by asterisks (* *p* < 0.05; ** *p* < 0.01; *** *p* < 0.001). Treatment groups: CK: Sterile water pretreatment 72 h prior to *N. laricinum* inoculation; JL54: JL54 bacterial suspension pretreatment 72 h prior to *N. laricinum* inoculation.

Malondialdehyde (MDA) content, an indicator of membrane lipid peroxidation, was significantly lower in JL54 seedlings compared with CK following pathogen inoculation (Figure 5a). At 12 hpi, MDA levels in the JL54 were reduced by 27.2% relative to the CK (16.18 ± 1.23 nmol/g fresh weight; FW vs. CK 22.23 ± 0.97 nmol/g FW; *p* < 0.001), while a moderate but significant reduction was also observed at 48 hpi (18.33 ± 1.31 nmol/g FW vs. CK 20.22 ± 0.25 nmol/g FW; *p* < 0.05).

Activities of key defense-related enzymes were markedly altered by JL54 pretreatment during *N. laricinum* infection (Figure 5b–d). POD activity in JL54-treated seedlings increased rapidly and was significantly higher than that in control plants at 6 hpi. (3038 ± 339.5 U/g vs. CK 1793.3 ± 322.7 U/g; *p* < 0.01; Figure 5b). PAL activity in JL54 seedlings consistently exceeded CK levels within 24 hpi. (Figure 5c). At 6 hpi, JL54 seedlings exhibited 50.3% higher activity than CK (848.5 ± 66.5 U/g vs. control 564.5 ± 122.7 U/g; *p* < 0.01); At 24 hpi, JL54 seedlings exhibited 24.2% higher activity than CK (901.9 ± 44.1 U/g vs. CK 725.9 ± 164.4 U/g; *p* < 0.05). GST activity exhibited distinct temporal patterns between treatments (Figure 5d). This enzymatic profile is consistent with the transcriptomic findings, where the glutathione metabolism pathway was highly enriched at 12 hpi (Figure 3f). In JL54 seedlings, GST activity peaked sharply at 12 hpi and was significantly higher than that in control plants (848.5 ± 66.5 U/g vs. 53.2 ± 10.1 U/g; *p* < 0.001). In contrast, CK seedlings displayed a delayed GST peak at 24 hpi. At this time point, GST activity in JL54 seedlings was significantly lower than in the CK group (62.1 ± 34.5 U/g vs. CK 449.7 ± 62.7 U/g; *p* < 0.001). This earlier and stronger GST induction in JL54 seedlings suggests a more rapid activation of detoxification pathways in response to pathogen challenge.

### 2.6. JL54 Modulates Endogenous Phytohormone Dynamics to Prime Systemic Resistance in L. olgensis

**Figure 6:**
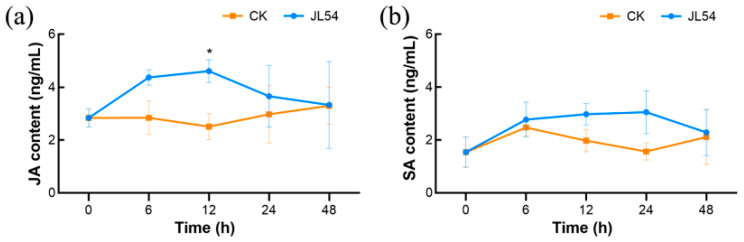
Jasmonic acid (JA) and salicylic acid (SA) accumulation in *Larix olgensis* needles pretreated with *Bacillus amyloliquefaciens* JL54 and challenged with *Neofusicoccum laricinum*: (**a**) JA content (ng/g fresh weight) at 0, 6, 12, 24, and 48 h post-inoculation. (**b**) SA content (ng/g fresh weight) at 0, 6, 12, 24, and 48 h post-inoculation. Data are means ± SE of three replicates. Significant differences between treatments are indicated by * p < 0.05 (ANOVA with LSD post hoc test). Treatment groups: CK: Sterile water pretreatment 72 h prior to *N. laricinum* inoculation; JL54: JL54 bacterial suspension pretreatment 72 h prior to *N. laricinum* inoculation.

Endogenous phytohormone levels in *L. olgensis* needles were monitored to elucidate the signaling pathways involved in JL54-induced resistance against *N. laricinum*. Both jasmonic acid (JA) and salicylic acid (SA) contents exhibited temporal fluctuations following JL54 treatment and pathogen inoculation (Figure 6). JA levels in JL54-colonized seedlings were consistently higher than controls throughout the infection period, with the most pronounced difference at 12 hpi. At this peak time point, JL54 seedlings exhibited 83.8% higher JA accumulation (4.61 ± 0.42 ng/g FW) compared to controls (2.51 ± 0.49 ng/g FW; *p* < 0.05). Thereafter, JA levels in JL54 seedlings gradually declined and converged toward control levels (Figure 6a). In contrast, SA showed no significant induction, with peak levels at 24 hpi (3.0546 ± 0.8 ng/g FW) statistically indistinguishable from controls (1.5672 ± 0.32301 ng/g FW; *p* > 0.05; Figure 6b). The JA burst coincided with the peak of GST activity at 12 hpi, suggesting that JA may act as a key signal to prime early defense responses in *L. olgensis* upon pathogen challenge. In contrast, the lack of significant SA induction indicated that SA-mediated systemic acquired resistance (SAR) was not evidently activated in this interaction system.

**Figure 7:**
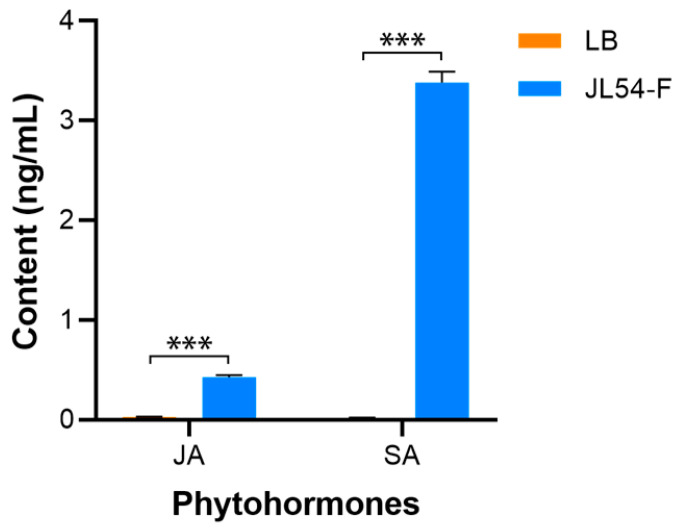
Jasmonic acid (JA) and salicylic acid (SA) detected in culture filtrates of *Bacillus amyloliquefaciens* JL54: Data are means ± SE of three biological replicates. Significant differences between treatments are indicated by asterisks (*** *p* < 0.001). Treatment groups: LB: The LB culture medium control; JL54-F: The sterile fermentation filtrate of JL54.

JA and SA were detected in the sterile fermentation filtrate of JL54 (JL54-F), whereas no detectable levels were observed in the LB medium (Figure 7). Quantitative analysis showed that the SA content in JL54-F was markedly higher than that of JA. Specifically, SA accumulated to 3.38 ng/mL, whereas JA was detected at a lower concentration of 0.43 ng/mL. These results indicate that JL54 fermentation filtrates contain measurable levels of phytohormones, with SA being more abundant than JA under the tested conditions.

## 3. Discussion

Larch shoot blight caused by *N. laricinum* represents a devastating disease causing significant ecological damage and economic losses in global forestry production. In this study, JL54 demonstrated a 43.75% control efficacy against *N. laricinum* in *L. olgensis* under controlled conditions, which aligns with previous reports of *Trichoderma atroviride* achieving no less than 44.69% efficacy against this pathogen [22].

Previously reported studies have shown that *B. amyloliquefaciens* PMB05 enhances reactive oxygen species (ROS) burst in strawberry during pathogen infection [23]. Similarly, we observed *B. amyloliquefaciens* JL54 triggered ROS production in *L. olgensis*, where H_2_O_2_ accumulation correlated strongly with systemic acquired resistance (SAR) activation. As a key ROS molecule, H_2_O_2_ not only directly inhibits pathogens but also activates downstream defense-related gene expression, inducing hypersensitive response (HR) and pathogenesis-related (PR) protein synthesis to establish early disease barriers [24,25]. Furthermore, *B. amyloliquefaciens* JL54 specifically promoted lignin biosynthesis in larch stems and programmed cell death (PCD) in needles, representing previously unreported defense strategies in this system. Lignin biosynthesis enhances physical barriers by strengthening cell wall integrity to restrict pathogen invasion [26], while its phenolic derivatives simultaneously disrupt pathogen enzyme activity, creating dual antimicrobial effects [24,27]. The observed PCD suggests potential hypersensitive response-mediated containment of pathogen spread [28]. This phenomenon reflects plants’ strategic balancing of disease resistance and growth through localized control of PCD [29]. The absence of significant callose deposition enhancement in JL54-treated groups versus pathogen-only groups suggests a coordinated defense strategy where plants balance callose metabolism with alternative pathways such as H_2_O_2_ burst and lignin deposition, rather than relying on singular mechanisms. Collectively, JL54 increases larch resistance to shoot blight by modulating H_2_O_2_ signaling, promoting lignin deposition, and inducing moderate PCD—physiological processes directly linked to physical barrier reinforcement and immune signal activation in plant defense systems.

GO annotation analysis revealed substantial differential gene expression in both biological processes and molecular functions across JL54-treated groups at 0 h and 12 hpi, indicating that JL54-primed *L. olgensis* employs more complex cellular activities and enhanced metabolic processes to mitigate pathogen damage. KEGG enrichment analysis demonstrated significant DEG accumulation in JL54-treated groups at 0 hpi within cutin/suberin/wax biosynthesis, phenylpropanoid biosynthesis, tryptophan metabolism, and nitrogen metabolism pathways. The enrichment in cutin, suberin, and wax biosynthesis pathways suggests JL54 may enhance plant stress resistance by strengthening physical barriers through cuticle thickening. Concurrently, phenylpropanoid biosynthesis activation (e.g., PAL genes) promotes lignin deposition to reinforce cell wall mechanical strength, providing certain structural support during growth [30]. At 12 hpi, JL54-treated groups showed significant DEG enrichment in ribosome pathways (40S/60S subunits) and endoplasmic reticulum protein processing (HSPs, GSTs), indicating potential resistance mechanisms through protein homeostasis maintenance and stress-damaged structure repair. Parallel to our findings, Wu’s team observed that preClpD precursor overaccumulation upregulated cytoplasmic ribosomal subunits, 26S proteasomes, and HSP family members (e.g., ClpB1/HOT1), which directly participate in misfolded protein degradation and refolding. Specifically, chaperone ClpB1/HOT1 resolves precursor protein aggregates (ChloroStore) to maintain cytoplasmic proteostasis, alleviating stress-induced leaf chlorosis and growth inhibition [31]. Artemisinin resistance studies revealed HSP90 binds misfolded proteins for clearance via ER-associated degradation (ERAD), underscoring HSPs’ central role in stress damage repair [32]. Collectively, JL54 likely enhances *L. olgensis* resistance against *N. laricinum* through early-stage phenylpropanoid activation for physical barriers and late-stage proteostasis/redox balance maintenance.

Malondialdehyde (MDA), a lipid peroxidation biomarker, reflects the extent of membrane damage [33,34]. While JL54 inoculation caused marginal MDA elevation, it significantly reduced MDA levels during pathogen challenge compared to untreated controls, demonstrating mitigated membrane oxidative damage. This aligns with reports that multiple *B. amyloliquefaciens* strains activate plant antioxidant defense systems, substantially decreasing MDA content in *Nicotiana benthamiana* while enhancing disease resistance [35]. Notably, JL54 inoculation induced earlier glutathione S-transferase (GST) activity elevation compared to controls. Since GST upregulation marks active cellular oxidative damage repair, this demonstrates JL54 primes *L. olgensis* for more proactive defense strategies against *N. laricinum* invasion. Remarkably, the sudden GST activity surge at 12 hpi in JL54-treated groups precisely coincided with the first observed malondialdehyde (MDA) content reduction, providing direct evidence that JL54 activates the host’s antioxidant defense system to mitigate membrane lipid peroxidation damage.

Notably, transcriptomic and RT-qPCR validation at 12 hpi revealed downregulated expression of several glutathione metabolism-related genes, while the GST enzyme activity in JL54-treated seedlings was significantly elevated at the same time point. This apparent inconsistency is presumably attributed to the multi-level complex regulation of GST:

First, GST constitutes a large multigene family in plants. The genes validated in this study only represent a subset of GST-related transcripts, and the downregulation of specific isoforms does not reflect the overall GST enzyme activity, which can be maintained or enhanced by the contribution of other family members. Second, enzyme activity is not directly correlated with transcript abundance, as disease resistance-related proteins can be functionally activated through post-transcriptional and post-translational mechanisms such as protein modification and RNA-mediated regulation. Similar discrepancies between gene expression and functional responses have been reported in transcriptomic studies of plant-insect interactions: defense-related proteins were activated without synchronous upregulation of their corresponding transcripts, confirming the existence of regulatory mechanisms beyond the transcriptional level [36].

In summary, the elevated GST activity observed in JL54-treated seedlings is more likely a reflection of the rapid biochemical activation of the antioxidant system, rather than the uniform upregulation of all glutathione metabolism-related genes at the transcriptional level.

POD serves as both a crucial reactive oxygen species (ROS)-scavenging enzyme and a documented inhibitor of MDA accumulation [37]. Simultaneously, POD functions as a secondary messenger activating early pathogen recognition systems while catalyzing lignin monomer polymerization into lignin polymers [38]. POD and PAL exhibited synchronous activity patterns in our study, with PAL serving as the upstream rate-limiting enzyme in lignin monomer biosynthesis. JL54-treated larches displayed significantly higher and earlier POD/PAL activity peaks compared to untreated controls. Consistent with our earlier findings of JL54-enhanced lignin deposition, this tripartite coordination (POD-PAL-lignin) indicates active lignin synthesis as a core defense strategy against *N. laricinum*, with JL54 effectively accelerating this protective process.

Salicylic acid (SA) serves as the essential systemic signal for systemic acquired resistance (SAR), whereas jasmonic acid (JA) mediates induced systemic resistance (ISR) [39]. *Arabidopsis* studies have demonstrated that the NPR1 protein acts as the central node connecting these pathways, with plants co-expressing both SAR and ISR exhibiting significantly enhanced resistance to subsequent *Pseudomonas syringae* pv. *tomato* infections compared to those activating either pathway alone [40]. The JL54 fermentation filtrate contained significantly elevated levels of both JA and SA compared to controls, suggesting its potential to activate SAR or ISR mechanisms in host plants through modulation of the JA-SA signaling network. In our study, although the SA content in the culture supernatants of JL54 was higher than that of JA, no significant SA accumulation was detected in the planta. This finding suggests that bacterium-derived SA may not be efficiently perceived by the host or successfully transduced into the host’s SA signaling pathway. Transcriptomic analysis revealed significant enrichment of pathways related to fatty acid metabolism, α-linolenic acid metabolism, phenylpropanoid biosynthesis, glutathione metabolism, and plant–pathogen interactions at the transcriptional level, particularly at the 12 h time point. Given that α-linolenic acid serves as the precursor for JA biosynthesis, and JA signaling is typically associated with the activation of phenylpropanoid metabolism, ROS regulation, and defense-related pathways, the transcriptomic results are generally consistent with the hormonal data. Collectively, these results indicate that JL54 may prime the host’s JA signaling pathway, enabling the plant to rapidly initiate early defense responses upon pathogen challenge. Regarding the discrepancy between the hormone levels in the fermentation filtrate and those in planta, we postulate that this may arise from the complexity of plant immune regulation. Bacterial metabolites are not direct determinants of hormone signaling; instead, the defense pathway activated in the host depends on pathogen challenge and is precisely modulated by signal crosstalk.

In summary, JL54 modulates a time-dependent and multi-layered defense response in *L. olgensis*, where transcriptomic reprogramming directly drives detectable physiological and biochemical changes. At 0 hpi, the enrichment of cutin/suberin/wax biosynthesis and phenylpropanoid biosynthesis pathways in the transcriptome was consistent with the enhanced lignin deposition observed in histochemical assays. The upregulation of PAL genes and the subsequent increase in PAL enzyme activity further supported the formation of this structural defense, collectively constructing a physical barrier against *N. laricinum* invasion. At 12 hpi, the enrichment of ribosome, endoplasmic reticulum protein processing and glutathione metabolism pathways was tightly correlated with biochemical data: the transcriptional upregulation of GST genes coincided with the sharp peak of GST activity at 12 hpi, which directly alleviated membrane lipid peroxidation (27.2% reduction in MDA content) and mitigated pathogen-induced cellular damage. Meanwhile, the moderate elevation of endogenous JA at 12 hpi (matching the peak of GST activity) suggested that the JA signaling pathway may act as a central node linking transcriptomic reprogramming and biochemical defense activation, priming the host for a rapid response to pathogen challenge.

While the present study elucidates the multi-layered defense mechanisms primed by *B. amyloliquefaciens* JL54 in *L. olgensis* against *N. laricinum* via integrative physiological, biochemical and transcriptomic analyses, several inherent limitations merit acknowledgment and further investigation. The transcriptomic profiling was only conducted at two time points (0 hpi and 12 hpi) post-pathogen inoculation, which restricts the characterization of dynamic and continuous transcriptional reprogramming throughout the entire infection process, particularly the late-stage regulatory networks associated with pathogen containment and host recovery. This study also focused exclusively on aboveground needle tissues, leaving the regulatory role of JL54 in root systems unexplored—especially the root-to-shoot signal transduction pathways that mediate systemic defense responses, also an important component of endophyte-induced plant resistance. Additionally, the specific molecular targets of JA signaling in JL54-primed defense remain uncharacterized; the crosstalk between JA and other phytohormones, including auxin and ethylene, in this conifer–endophyte–pathogen interaction system has not been clarified, and the potential synergistic or antagonistic effects of these phytohormone pathways on resistance induction await verification. Furthermore, transcriptomic data implied the involvement of tryptophan metabolism in JL54-mediated resistance, which may be linked to auxin biosynthesis [41], yet whether this pathway and other uncharacterized metabolic pathways contribute to defense priming remains to be further validated through metabolomic profiling and functional gene assays such as gene silencing or overexpression.

## 4. Materials and Methods

### 4.1. Plant Material and Growth Conditions

Seeds and one-year-old seedlings of *L. olgensis* were obtained from the Maohui Nursery in Dunhua, Jilin Province, China. Prior to sowing, seeds were surface-sterilized by immersion in 75% (*v*/*v*) ethanol for 30 s, followed by treatment with 30% (*v*/*v*) hydrogen peroxide for 20 min, and subsequently rinsed three times with sterile distilled water. Sterilized seeds were sown in a growth substrate consisting of peat, vermiculite, and perlite (2:1:1, *v*/*v*/*v*), which had been autoclaved at 121 °C for 2 h. Seedlings were cultivated in plastic pots (8.5 cm height × 8.2 cm top diameter × 5.1 cm bottom diameter) and maintained in a controlled-environment growth chamber at 25 ± 1 °C under a 16 h light/8 h dark photoperiod, with a relative humidity of approximately 70%.

One-year-old seedlings were directly transplanted into pots containing the same sterilized substrate mixture and grown under identical environmental conditions.

### 4.2. Microbial Strains and Culture Conditions

*B. amyloliquefaciens* JL54 (deposited at the China Center for Type Culture Collection, Wuhan, China; accession number M 2023793) was isolated from healthy larch branches and leaves collected in Dunhua, Jilin Province, China. For inoculation experiments, strain JL54 was cultured in Luria–Bertani (LB) liquid medium at 37 °C with shaking at 200 rpm for 24 h. Bacterial cells were harvested by centrifugation at 10,000× *g* for 15 min and resuspended in sterile distilled water to a final concentration of approximately 1 × 10^8^ CFU/mL, as determined using the dilution plate method. The sterile fermentation filtrate of JL54 was obtained by centrifugation followed by filtration through a 0.22-μm membrane.

The larch shoot blight pathogen *N. laricinum* (strain DHKS 6-3), provided by the Jilin Academy of Forestry Sciences, was originally isolated from diseased larch trees in Dunhua. The pathogen was cultured on potato dextrose agar (PDA) medium at 25 °C, and 6 mm-diameter mycelial plugs excised from the actively growing margins of colonies were used for inoculation.

### 4.3. Experimental Design and Inoculation Procedures

For induced resistance assays, *L. olgensis* seedlings in the treatment group were inoculated with 10 mL of *B. amyloliquefaciens* JL54 suspension (1 × 10^8^ CFU/mL), whereas control plants received an equal volume of sterile water. After 72 h, all seedlings were inoculated with *N. laricinum* using the wound-bark attachment method. Briefly, superficial wounds were created on seedling stems with sterile syringe needles, and 6 mm-diameter mycelial plugs of *N. laricinum* prepared as described in Section 4.2 were attached to the wound sites. No spore suspension was used in this study. All inoculated plants were maintained in sealed humidification bags for 72 h, and inoculations were conducted under consistent environmental conditions to ensure the reproducibility of disease index assessment. Each treatment was conducted with 8 biological replicates. Disease incidence and severity indices were subsequently assessed once disease symptoms were fully developed in control plants.

Disease severity was classified into 5 grades based on the proportion of diseased needles: Grade 0: No visible disease symptoms; Grade 1: Less than 25% of needles showing disease symptoms; Grade 2: 25% to 50% of needles showing disease symptoms; Grade 3: 50% to 75% of needles showing disease symptoms; Grade 4: More than 75% of needles showing disease symptoms, including nearly dead seedlings.

The calculation formulas of disease incidence, disease index, and control efficacy are as follows:

(1) $$Disease\;Incidence=\left(number\;of\;diseased\;plants∕\;total\;number\;of\;investigated\;plants\right)\times 100\%,$$

(2) $$Disease\;Index=\left\{\left[\sum \left(representative\;value\;of\;disease\;grade\times \;number\;of\;diseased\;plants\;at\;each\;grade\right)\right]∕\;\left(representative\;value\;of\;the\;highest\;level\times \;total\;number\;of\;investigated\;plants\right)\right\}\times 100\%,$$

(3) $$Control\;Efficacy=\left[\left(disease\;index\;of\;negative\;control\;group-\;disease\;index\;of\;treatment\;group\right)∕disease\;index\;of\;negative\;control\;group\right]\times \;100\%.$$

For histochemical staining, transcriptome sequencing, and physiological analyses, distinct sampling strategies were applied. Stem and needle tissues were collected at 48 hpi for histochemical examination. Apical needles from three randomly selected seedlings per treatment were harvested at 0 and 12 hpi for transcriptomic analysis. Physiological assays were conducted using samples collected at 0, 6, 12, 24, and 48 hpi. All samples were immediately frozen in liquid nitrogen and stored at −80 °C until further analysis. Each treatment and control group included three biological replicates, with each replicate derived from an independent seedling individual.

### 4.4. Physiological and Biochemical Assays

#### 4.4.1. Histochemical Assays for Induced Resistance

Needle and stem tissues collected at 48 hpi were subjected to histochemical staining to visualize defense-related responses. H_2_O_2_ accumulation was detected using 3,3′-diaminobenzidine (DAB) staining, with brown precipitates indicating H_2_O_2_ localization [42]. Lignin deposition in stem tissues adjacent to inoculation sites was examined by phloroglucinol-HCl staining, where red coloration represented lignin accumulation [42]. Callose deposition in needle tissues was visualized by aniline blue fluorescence staining and observed under a Zeiss fluorescence microscope (excitation 390 nm, emission 460 nm), with blue fluorescence indicating callose presence [43]. Cell death was assessed using trypan blue staining, with blue-stained cells indicating nonviable tissue [44].

#### 4.4.2. Physiological and Biochemical Assays for Induced Resistance Mechanisms

To characterize JL54-induced resistance mechanisms, malondialdehyde (MDA) content (nmol/g FW) and activities of peroxidase (POD), phenylalanine ammonia-lyase (PAL), and glutathione S-transferase (GST) (U/g FW) were quantified using commercial assay kits (Nanjing Z&Y Biotechnology Co., Ltd., Nanjing, China). Endogenous phytohormone levels in needles, including jasmonic acid (JA) and salicylic acid (SA), were determined using plant-specific ELISA kits for jasmonic acid and salicylic acid (Nanjing Z&Y Biotechnology Co., Ltd., China), according to the manufacturer’s instructions. Absolute concentrations of jasmonic acid (JA) and salicylic acid (SA) of JL54 fermentation filtrates and LB medium controls were determined by high-performance liquid chromatography-tandem mass spectrometry (HPLC-MS/MS) (Nanjing Rayor Biotechnology Co., Ltd., Nanjing, China).

### 4.5. RNA Extraction and Transcriptome Sequencing

Total RNA was extracted from frozen needle samples using the OminiPlant RNA Kit (DNase I) (Jiangsu Cowin Biotech Co., Ltd., Taizhou, China). RNA concentration and purity were assessed using a NanoDrop One spectrophotometer (Thermo Fisher Scientific, Waltham, MA, USA), and integrity was evaluated by agarose gel electrophoresis and Agilent 5300 analysis. Samples with RNA integrity number (RIN) > 8.0 and RNA quality number (RQN) > 9.0 were used for library construction.

cDNA libraries were constructed and sequenced on the NovaSeq X Plus platform (paired-end, 150 bp) by Shanghai Majorbio Bio-pharm Technology Co., Ltd, Shanghai, China. Raw reads were filtered for quality, followed by de novo assembly using Trinity (v2.8.5), optimization with TransRate (v1.0.3), redundancy removal with CD-HIT, and completeness assessment using BUSCO (v3.0.2).

Differentially expressed genes (DEGs) were identified using DESeq2 (v1.42.0) with thresholds of |log_2_FC| ≥ 1 and adjusted *p*-value < 0.05 (Benjamini–Hochberg correction). Functional annotation was performed against public databases (NR, Swiss-Prot, Pfam, and eggNOG databases), Gene Ontology (GO) enrichment was conducted using Goatools, and KEGG pathway analysis was carried out with BH-adjusted *p*-values.

KEGG enrichment analysis was performed using the Majorbio Cloud Platform. Rich Factor was calculated as the number of differentially expressed genes (DEGs) annotated to a pathway divided by the total number of genes annotated to that pathway in the *Larix olgensis* reference transcriptome. Rich Factor was calculated as:

Rich Factor = (Number of DEGs annotated to the pathway)/(Total number of genes annotated to the pathway in the *Larix olgensis* reference transcriptome). Pathways with Padjust < 0.05 were considered significantly enriched.

Six randomly selected DEGs, including defense-related genes (PAL and GST), were validated by RT-qPCR using *LoB80280* (NCBI accession No. MF278617.1) as the internal reference gene. De novo transcriptome assembly was performed using Trinity software (v2.8.5), and *LoB80280* was selected as the reference gene [45]. The RT-qPCR analysis was performed using samples from the JL54 treatment and CK control groups 12 h after pathogen inoculation. Relative expression levels were calculated using the 2^−ΔΔCt^ method. Primer sequences are listed in Table 2. All sequencing data have been deposited in the NCBI Sequence Read Archive (SRA).

### 4.6. Statistical Analyses

All experiments were conducted with at least three biological replicates. Data are presented as means ± standard error (SE). Statistical analyses and graphical visualization were performed using GraphPad Prism 9. One-way analysis of variance (ANOVA) followed by least significant difference (LSD) tests was applied, and differences were considered statistically significant at *p* < 0.05.

Histochemical staining images were captured using Zeiss microscopy systems, and staining intensities were quantified using ImageJ software (v1.53). Statistical analyses for histochemical data followed the same procedures applied to physiological measurements.

## 5. Conclusions

This study reveals that *B. amyloliquefaciens* JL54 enhances *L. olgensis* resistance against *N. laricinum* through multi-layered, synergistic mechanisms. JL54 not only triggers early-stage H_2_O_2_ burst, phenylpropanoid metabolism, and lignin deposition to strengthen physical and chemical barriers, but also mitigates oxidative damage and cellular structural disruption through PCD regulation and late-stage proteostasis maintenance via upregulated heat shock proteins and GSTs. Furthermore, JL54 may modulate the host’s endogenous phytohormone dynamics, with a moderate elevation of JA potentially contributing to the priming of early defense signaling, which aids in the establishment of host resistance against shoot blight.

## Figures and Tables

**Table 1:** Disease incidence and control efficacy of *Bacillus amyloliquefaciens* JL54 in *Larix olgensis* seedlings.

Treatment	Disease Incidence (%)	Disease Index	Control Efficacy (%)
JL54 suspension	87.5	45	43.75
Sterile water (CK)	100	80	-

**Table 2:** Gene selection and primer design for qRT-PCR validation.

Gene ID	Forward Primer (5′–3′)	Reverse Primer (5′–3′)
DN1238_c0_g1	GAGAGCAGCAACAGAAAGCG	CCACCCAATGCGATGTCAAG
DN2582_c0_g3	CGAGCTCGTCTTCGTCGATT	GCTCTCGACTCGAACAAGGT
DN3787_c0_g1	TAGCGTCAGAAGGAAGCACG	GAAAGCGCGAGGAAAACGAG
DN4092_c0_g1	CCGCATGTCCTTAACCAGAGT	CCGCATGTCCTTAACCAGAGT
DN8804_c0_g1	CGAGGGATCTTGGCCACTTT	GCCGTCTTCCACCTCAATCT
DN32708_c0_g1	AGGCTGCTATTTTGAGCGGT	TCTTTCTTGGCGGGAATCGT
LoB80280	GCCGTGCTGCTGGATAATGAGG	TGTCTGGAACTCAGTCACATCAACG

## Data Availability

The RNA sequencing data generated in this study have been deposited in the NCBI Sequence Read Archive (SRA) under accession number PRJNA1378436, available at http://www.ncbi.nlm.nih.gov/bioproject/1378436 (accessed on 7 April 2026).

## Abbreviations

The following abbreviations are used in this manuscript:

PAMP	Pathogen-associated molecular pattern
PRR	Pattern-recognition receptor
PTI	PAMP-triggered immunity
RNA	Ribonucleic acid
H_2_O_2_	Hydrogen peroxide
PCD	Programmed cell death
DEG	Differentially expressed gene
hpi	Hour post-inoculation
GO	Gene Ontology
KEGG	Kyoto Encyclopedia of Genes and Genomes
PAL	Phenylalanine ammonia-lyase
POD	Peroxidase
HSP	Heat shock protein
GST	Glutathione S-transferase
RT-qPCR	Reverse transcription quantitative polymerase chain reaction
FW	Fresh weight
MDA	Malondialdehyde
JA	Jasmonic acid
SA	Salicylic acid
ISR	Induced systemic resistance
SAR	Systemic acquired resistance
